# Diagnostic value of serum TGF-β1 and CysC in type 2 diabetic kidney disease: a cross-sectional study

**DOI:** 10.3389/fmed.2025.1529648

**Published:** 2025-04-11

**Authors:** Yi Kang, Qian Jin, Mengqi Zhou, Huijuan Zheng, Xiaobin Li, Aoshuang Li, Jing Wei Zhou, Jie Lv, Yaoxian Wang

**Affiliations:** ^1^Dongzhimen Hospital, Beijing University of Chinese Medicine, Beijing, China; ^2^Renal Research Institution of Beijing University of Chinese Medicine, Beijing, China; ^3^Graduate School of Beijing University of Chinese Medicine, Beijing, China; ^4^Department of Traditional Chinese Medicine, Beijing Puren Hospital, Beijing, China

**Keywords:** type 2 diabetes, diabetic kidney disease, TGF-β1, CysC, diagnostic value

## Abstract

**Background:**

Diabetic kidney disease (DKD) is one of the common microvascular complications of diabetes. The exploration of serum biomarkers holds promise for improving the efficiency and accuracy of early DKD diagnosis. This study aims to investigate the diagnostic value of transforming growth factor-β1 (TGF-β1) and cystatin C (CysC) in DKD patients.

**Methods:**

A total of 126 patients with type 2 diabetes mellitus (T2DM) diagnosed at Dongzhimen Hospital, Beijing University of Chinese Medicine, between May 2021 and March 2023 were enrolled. Patients were categorized based on proteinuria levels and estimated glomerular filtration rate (eGFR). Correlation analyses were conducted to examine the relationships between serum TGF-β1, CysC, and clinical parameters. Logistic regression was applied to identify correlation factors for DKD and renal function impairment in T2DM patients. Furthermore, receiver operating characteristic (ROC) curve analysis was performed to assess diagnostic efficacy.

**Results:**

Significant differences in TGF-β1 and CysC levels were observed across groups with varying proteinuria levels. CysC was positively correlated with TGF-β1 (*r* = 0.640, *p* < 0.001). TGF-β1 has been associated with proteinuria levels in T2DM patients. Each unit increase in TGF-β1 was associated with a 1.122-fold and 1.470-fold higher odds of the presence of microalbuminuria and proteinuria, respectively, in the normal proteinuria (NP) group. TGF-β1 and CysC showed varying diagnostic performance. TGF-β1 better distinguished microalbuminuria group (MP) from NP, while CysC alone was less effective. T2DM patients with impaired renal function exhibited significantly higher CysC and TGF-β1 levels compared to those with normal renal function. CysC emerged as an associated factor of renal function decline (OR = 2.255, *p* = 0.008). CysC demonstrated superior diagnostic efficacy compared to TGF-β1 in predicting renal function impairment (AUC = 0.974).

**Conclusion:**

CysC and TGF-β1 can serve as potential biomarkers for assessing renal impairment and proteinuria in T2DM patients. Their combined evaluation demonstrates diagnostic value and clinical application potential.

## 1 Introduction

Diabetes mellitus is a chronic metabolic disorder. According to the IDF Diabetes Atlas, type 2 diabetes mellitus (T2DM) accounts for more than 90% of all diabetes cases globally, with an incidence rate that continues to rise annually (1). Diabetic kidney disease (DKD) is one of the primary microvascular complications of T2DM and has become the leading cause of end-stage renal disease (ESRD) (2). Early diagnosis is critical for the prevention and treatment of DKD (3). However, the current gold standard of renal biopsy is invasive, non-replicable, and carries a risk of bleeding, making it unnecessary for clinical DKD diagnosis. Glomerular filtration rate (GFR) and proteinuria are key parameters for evaluating kidney function and the severity of chronic kidney disease (CKD). However, these changes are not specific to DKD. Moreover, GFR calculated based on serum creatinine levels often remains unremarkable in the early stages of DKD (2, 4). The sensitivity and accuracy of microalbuminuria in assessing renal disease onset and progression in T2DM patients have also been increasingly questioned (5–7). This often results in delayed diagnosis and intervention for DKD, and once overt nephropathy develops, it irreversibly progresses to ESRD. Therefore, researchers have been seeking new biomarkers for DKD to improve early diagnostic accuracy and enhance the ability to predict disease progression.

DKD lesions may involve the glomeruli, tubules, interstitium, and vasculature, ultimately leading to irreversible renal fibrosis. Transforming growth factor-beta1 (TGF-β1) is a recognized profibrotic factor involved in glomerulosclerosis, tubulointerstitial fibrosis, and tubular epithelial cell transdifferentiation, playing a crucial role in the progression of CKD (8). Under DKD pathological conditions, factors such as hyperglycemia and renin-angiotensin system (RAS) activation stimulate the production of TGF-β1 in tubular cells, podocytes, mesangial cells, and glomerular endothelial cells (9–12). TGF-β1 has been identified as a key pathogenic factor in DKD progression (13). Serum cystatin C (CysC), unaffected by race or sex, is emerging as a potential alternative to serum creatinine for assessing kidney function (14). However, some studies suggest that its utility in early DKD evaluation remains controversial (7, 15–17). Additionally, elevated CysC expression triggered by TGF-β1 appears to be a common feature of the fibrotic process (18). Meta-analyses of several randomized controlled trials have confirmed that increased serum levels of TGF-β1 and CysC are associated with an elevated risk of DKD (19, 20), highlighting their significance in the onset and progression of DKD. However, systematic studies on the diagnostic value of TGF-β1 and CysC in DKD patients and their correlation with clinical parameters remain limited.

This study aims to investigate the expression characteristics of TGF-β1 and CysC in patients with varying degrees of renal impairment, analyze their correlation with clinical parameters, and evaluate their diagnostic value. This provides new insights and evidence for early diagnosis and monitoring of DKD. Furthermore, by analyzing the relationship between these biomarkers and disease progression, this study seeks to deepen understanding of disease mechanisms and identify potential therapeutic targets.

## 2 Methods

### 2.1 Study design and population

**Figure 1:**
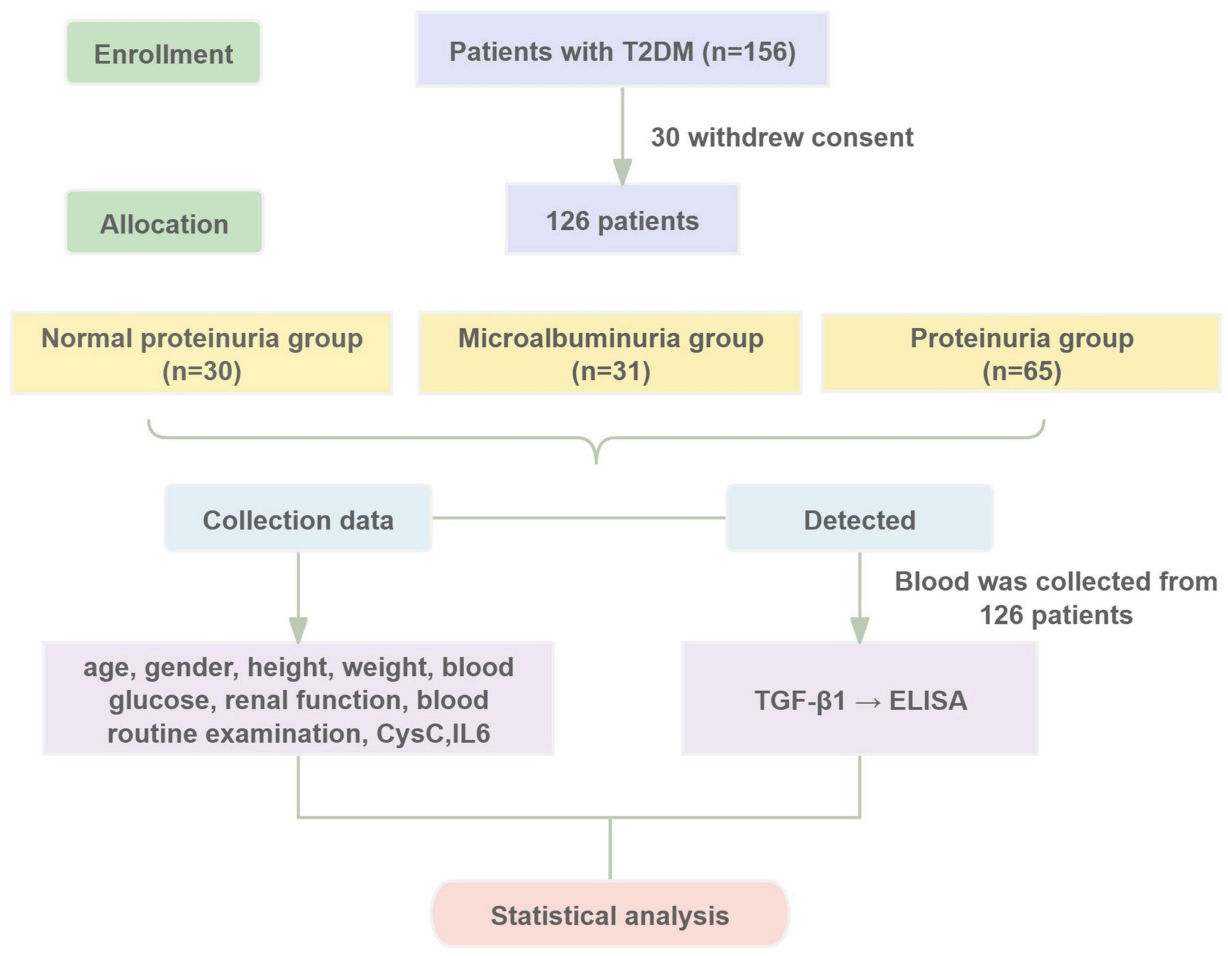
Diagram of the study design.

Patients diagnosed with T2DM at Dongzhimen Hospital, Beijing University of Chinese Medicine, from May 2021 to March 2023 were selected as study subjects. The study was approved by the Ethics Committee of Dongzhimen Hospital, Beijing University of Chinese Medicine (Approval No. 2022DZMEC-062-03), and informed consent was obtained from all participants. The study procedures are detailed in Figure 1.

### 2.2 Inclusion and exclusion criteria

Inclusion criteria: (1) Age between 30 and 90 years. (2) Diagnosis of T2DM based on the Guidelines for the Prevention and Treatment of Type 2 Diabetes in China (2020 Edition) (21); diagnosis of DKD based on the 2007 National Kidney Foundation (NKF-KD/OQI) Guidelines (22), the 2020 Clinical Practice Guideline for the Evaluation and Management of CKD by the Kidney Disease: Improving Global Outcomes (KDIGO) organization (23) and the 2021 Chinese Clinical Guidelines for the Diagnosis and Treatment of Diabetic Kidney Disease (24). (3) Availability of complete clinical data. (4) Voluntary signing of an informed consent form.

Exclusion criteria: (1) Patients who had undergone dialysis or kidney transplantation. (2) Patients with primary or secondary nephropathy. (3) Patients with urinary tract infections or other acute or chronic inflammatory conditions. (4) Patients with abnormal liver function, autoimmune diseases, malignancies, hematologic disorders, or mental illnesses. (5) Patients who had experienced trauma, surgery, or psychological stress within the past 6 months.

### 2.3 Grouping strategies

#### 2.3.1 Grouping strategy 1

The T2DM patients were categorized into three distinct groups based on their urinary albumin-to-creatinine ratio (UACR), which serves as an indicator of the severity of albuminuria. The groups included: the normal proteinuria group (NP group) (UACR <30 mg/g, *n* = 30), the microalbuminuria group (MP group) (UACR ranging from 30 to 300 mg/g, *n* = 31), and the proteinuria group (P group) (UACR >300 mg/g, *n* = 65).

#### 2.3.2 Grouping strategy 2

In a second classification, patients with T2DM were divided into two categories according to their estimated glomerular filtration rate (eGFR). These were: the normal renal function group (NRF group) (eGFR ≥90 mL/min/1.73 m^2^, *n* = 44) and the group with decreased renal function (DRF group) (eGFR <90 mL/min/1.73 m^2^, *n* = 82).

### 2.4 Data collection

#### 2.4.1 Participant characteristics

Clinical data were collected, including age, gender, height, weight, systolic blood pressure (SBP), and diastolic blood pressure (DBP). Body mass index (BMI) was calculated as weight divided by the square of height (kg/m^2^).

#### 2.4.2 Laboratory testing

All patients were required to fast for more than 8 h. A single venous blood sample was collected from each patient on the second day after admission, in the early morning, after fasting. The blood sample was processed for centrifugation within 2 h of collection. Key biochemical parameters, including fasting plasma glucose (FPG), total protein (TP), urea, serum creatinine (Scr), uric acid (UA), and CysC, were quantified using an automated biochemical analyzer (Beckman Coulter, AU5821, United States). Complete blood count (CBC) was performed using an automated hematology analyzer (Beckman Coulter, DXH900, United States). The level of interleukin-6 (IL-6) was measured by chemiluminescence assay with an electrochemical luminescence assay (Roche Diagnostics, cobas e401). Five milliliters morning urine sample was collected, and UACR was measured using an automated biochemical analyzer (Beckman Coulter, AU5800, United States) by nephelometric immunoassay.


$$\begin{array}{l} \mathrm{Systemic immune-inflammation index}\;\left(SII\right)\\ =\frac{\mathrm{Platelet count}*\mathrm{Neutrophil count}\;}{\mathrm{Lymphocyte count}}\text{.} \end{array}$$

#### 2.4.3 TGF-β1 measurement

Fasting venous blood samples were collected from all participants early on the second morning after admission and placed in anticoagulant tubes. The samples were centrifuged at 3,000 r/min at 4°C for 10 min, and the supernatant serum was aliquoted into EP tubes, labeled, and stored at −80°C. The TGF-β1 ELISA Kit (Wuhan Elabscience Biotechnology Co., Ltd., E-EL-0162) was used to determine the TGF-β1 concentration. The procedure was strictly followed according to the instructions. The intra-assay and inter-assay coefficients of variation were both <10%. For each plate, a standard curve (0–2,000 pg/mL, *R*^2^ > 0.99) and dual blank controls were set. The detailed operational procedure is provided in the Supplementary material.

### 2.5 Statistical analysis

All statistical analyses were performed using SPSS 26.0. The normality distribution was assessed using the Shapiro–Wilk test (*n* < 50) or the Kolmogorov–Smirnov test (*n* ≥ 50). *p* > 0.05 and the Q–Q plot indicates an approximate normal distribution, the data is considered to follow a normal distribution. For normally distributed data, comparisons between two groups were conducted using independent sample *t*-tests, and comparisons among multiple groups were performed using one-way analysis of variance (ANOVA). Non-normally distributed data were analyzed using nonparametric tests, with comparisons between two groups conducted using the Mann–Whitney *U* test and comparisons among multiple groups using the Kruskal–Wallis test. Categorical data were analyzed using the chi-square test. Bonferroni correction was applied to adjust *p*-values for multiple comparisons within groups. Pearson or Spearman rank correlation tests were used to assess relationships. Logistic regression analysis was conducted to identify correlated factors for DKD in T2DM patients. The area under the receiver operating characteristic (ROC) curve (AUC) was used to evaluate the diagnostic value. *p* < 0.05 was considered statistically significant.

## 3 Results

### 3.1 Analysis based on proteinuria groups

#### 3.1.1 Baseline characteristics of patients by different levels of proteinuria

**Figure 2:**
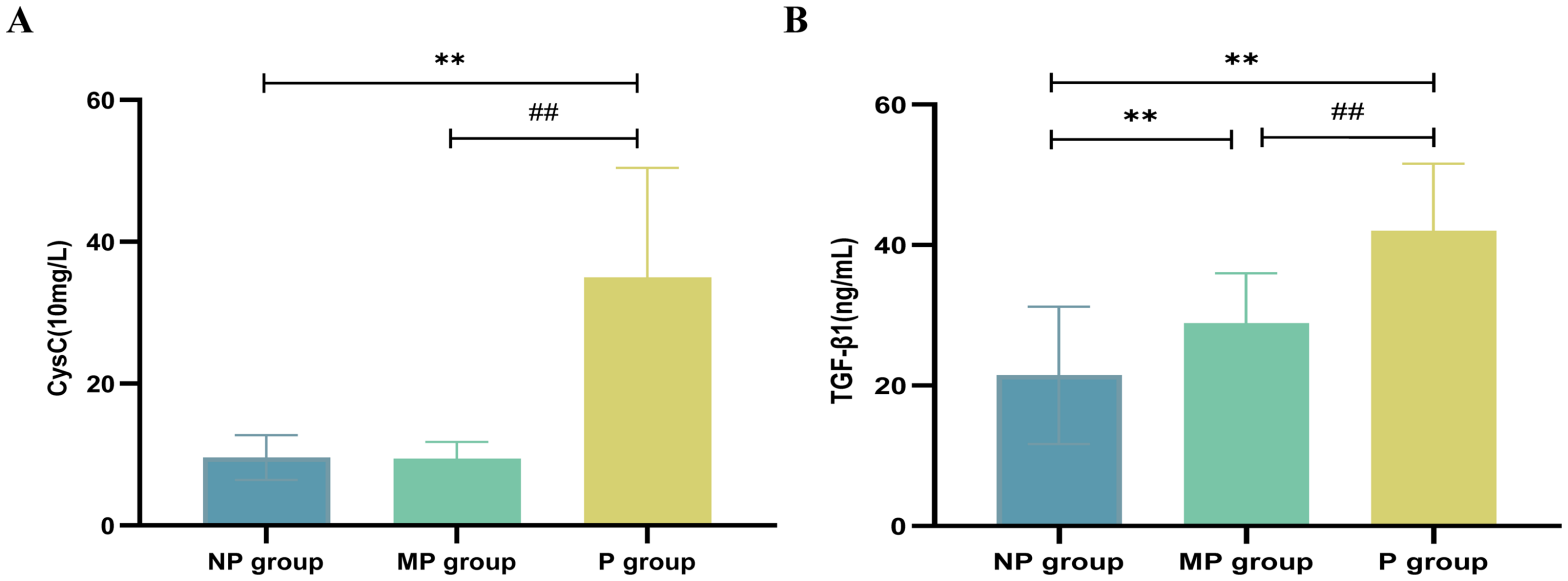
Expression levels of CysC and TGF-β1. **(A)** CysC. **(B)** TGF-β1. Compared with NP group, ^*^p < 0.05 and ^**^p < 0.01; compared with MP group, ^#^*p* < 0.05 and ^##^*p* < 0.01.

A total of 126 T2DM patients were enrolled in this study and classified into groups based on proteinuria levels: NP group (*n* = 30), MP group (*n* = 31), and P group (*n* = 65). No statistically significant differences were observed among the three groups in terms of age, gender, BMI, or DBP (*p* > 0.05). Significant differences in laboratory parameters, including Hb, PLT, FPG, UA, TP, Scr, and eGFR, were found among the three groups. Particularly, UA, Scr, and 24-UTP levels in the P group were significantly higher than other two groups (*p* < 0.001). Furthermore, SII, IL-6, and UACR showed highly significant differences among the three groups (*p* < 0.001) (Table 1). Serum CysC concentrations in each group were as follows: NP group, 9.583 ± 3.150 (10 mg/L); MP group, 9.410 ± 2.373 (10 mg/L); P group, 34.98 ± 15.46 (10 mg/L). Significant differences in serum CysC concentrations were observed among the three groups (*p* < 0.001), with CysC levels in the P group significantly higher than those in the NP and MP groups (*p* < 0.001) (Figure 2A). Serum TGF-β1 concentrations were as follows: NP group, 21.455 ± 9.790 ng/mL; MP group, 28.881 ± 7.115 ng/mL; P group, 42.041 ± 9.532 ng/mL. Significant differences in TGF-β1 concentrations were detected among the three groups (*p* < 0.001) (Figure 2B).

**Table 1:** Baseline characteristics of patients grouped by proteinuria levels.

Variable	NP group (*n* = 30)	MP group (*n* = 31)	P group (*n* = 65)	*p*-value
	NDKD (*n* = 30)	DKD (*n* = 96)		
Age (years)	58.8 ± 11.616	58.87 ± 11.575	60.12 ± 11.145	0.817
Gender				0.213
Male (%)	17 (56.67)	17 (54.84)	46 (70.77)	
Female (%)	13 (43.33)	14 (45.16)	19 (29.23)	
Durations (years)	10.47 ± 7.09	12.75 ± 7.16	17.38 ± 6.90	<0.001
BMI (kg/m^2^)	24.93 (22.69, 27.235)	25.54 (23.63, 29.76)	25.56 (22.97, 28.06)	0.798
SBP (mmHg)	134.8 ± 18.357	132.1 ± 18.846	144.02 ± 18.785^*##^	0.007
DBP (mmHg)	81.03 ± 14.371	78.13 ± 14.435	77.25 ± 10.364	0.388
Hb (g/L)	145.17 ± 15.757	140.35 ± 21.827	105.15 ± 20.136^**##^	<0.001
PLT (10^9^/L)	224.13 ± 49.309	236.39 ± 63.964^**^	235.52 ± 79.082^**##^	0.262
FPG (mmol/L)	10.825 (8.3825, 15.855)	10.07 (7.06, 14.49)	7.82 (6.345, 11.13)^**^	0.006
UREA (mmol/L)	5.67 (5.10, 6.86)	5.59 (4.42, 7.48)	11.53 (8.42, 14.70)6^**##^	<0.001
UA (μmol/L)	342.05 (284.35, 410.475)	295.4 (266.8, 407.4)	396 (329.7, 434.425)^**##^	<0.001
TP (g/L)	71.35 (66.25, 76.55)	73.3 (65.8, 75.3)	63.75 (59.325, 66.875)^**##^	<0.001
Scr (μmol/L)	63.25 (53.4, 83.15)	65.4 (60.7, 73)	334.6 (133.7, 476.8)^**##^	<0.001
eGFR (mL/min/1.73m^2^)	98.33 (83.83, 106.88)	97.25 (85.28, 116.87)	39.72 (22.57, 54.25)^**##^	<0.001
IL6 (pg/mL)	2.85 (1.5, 4.77)	2.98 (1.55, 5.78)	5.31 (3.53, 7.26)^**##^	<0.001
SII	488.84 (345.93, 790.99)	491.17 (398.47, 715.83)	595.82 (491.74, 957.48)	0.002
24-UTP (mg)	96.5 (51, 139)	174 (114, 246)	3,754 (1567.25, 6,861)^**##^	<0.001
UACR (mg/g)	15 (10, 16.25)	150 (100, 180)	800 (800, 1,500)^**##^	<0.001

BMI, body mass index; SBP, systolic blood pressure; DBP, diastolic blood pressure; WBC, white blood cell count; Hb, hemoglobin; PLT, platelet count; FPG, fasting plasma glucose; UA, uric acid; TP, total protein; Scr, serum creatinine; eGFR, estimated glomerular filtration rate; IL6, interleukin-6; SII, systolic index of inflammation; CysC, cystatin C; 24-UTP, 24-h urinary total protein; UACR, urine albumin creatinine ratio; TGF-β1, transforming growth factor beta 1. Compared with normal proteinuria group, ^*^p < 0.05 and ^**^p < 0.01; compared with microproteinuria group, ^#^*p* < 0.05 and ^##^*p* < 0.01.

#### 3.1.2 Correlation of TGF-β1 and CysC with clinical indicators

**Figure 3:**
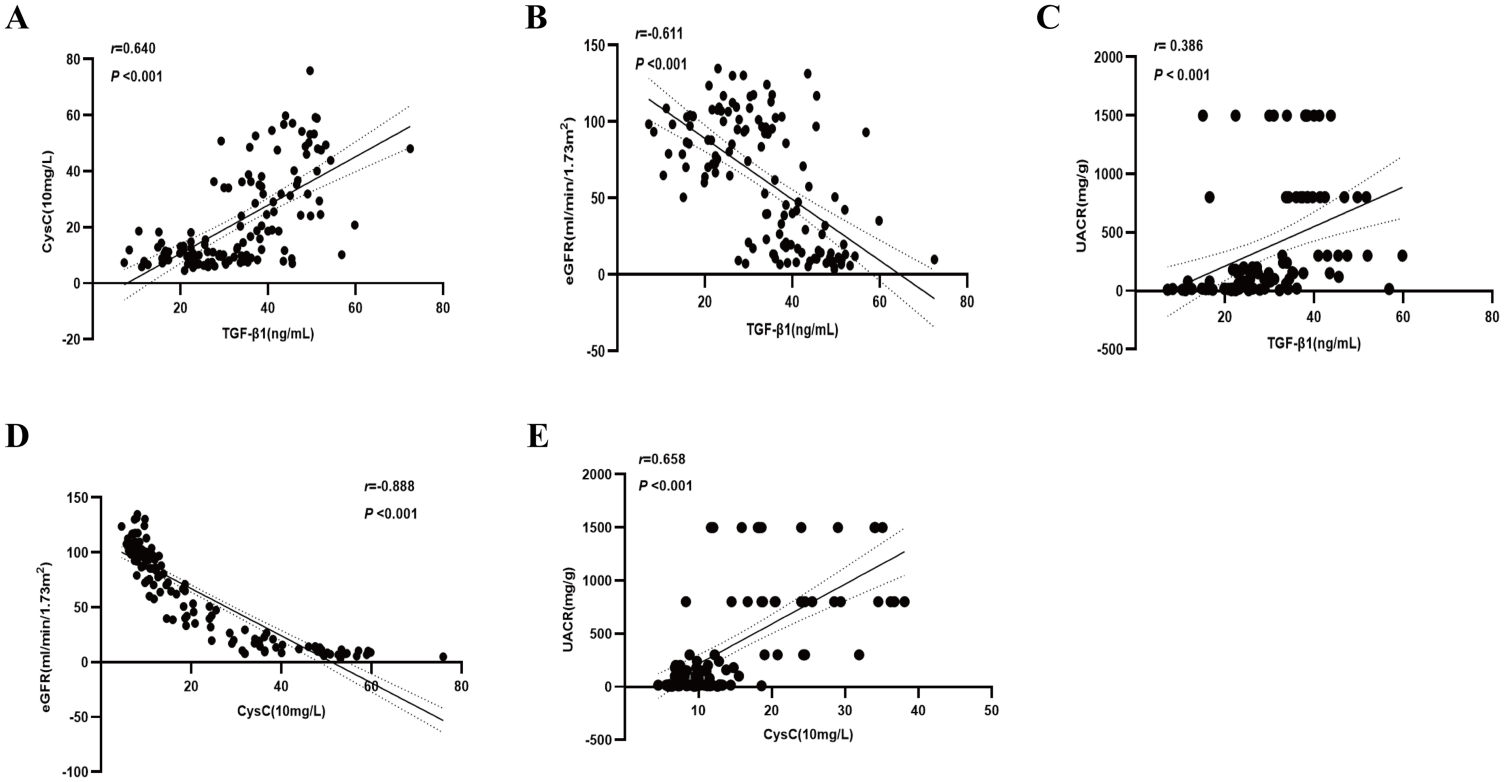
Correlation of TGF-β1 and CysC with clinical indicators. **(A)** Correlation between TGF-β1 and CysC. **(B)** Correlation between TGF-β1 and eGFR. **(C)** Correlation between TGF-β1 and UACR. **(D)** Correlation between CysC and eGFR. **(E)** Correlation between CysC and UACR.

Serum levels of TGF-β1 and CysC demonstrated complex correlations with multiple physiological markers. TGF-β1 exhibited significant positive associations with CysC (*r* = 0.640) (Figure 3A). TGF-β1 exhibited significant negative correlations with eGFR (*r* = −0.611; Figure 3B), age, Hb, and TP, while demonstrating positive correlations with 24-UTP (*r* = 0.507), UACR (*r* = 0.386; Figure 3C), PLT, UA, IL-6, SII, and SBP (Table 2). Multivariate linear regression analysis incorporating these covariates revealed that TGF-β1 remained significantly associated with PLT, 24-UTP, and UACR (Table 2). Similarly, CysC demonstrated strong positive correlations with 24-UTP (*r* = 0.585), UACR (*r* = 0.658; Figure 3E), IL-6, SII, UREA, UA, and SBP, along with significant inverse correlations with eGFR (*r* = −0.888; Figure 3D), FPG, Hb, and TP. Multivariate regression analysis adjusting for these variables confirmed that CysC maintained significant associations with eGFR and UACR (Table 3).

**Figure 4:**
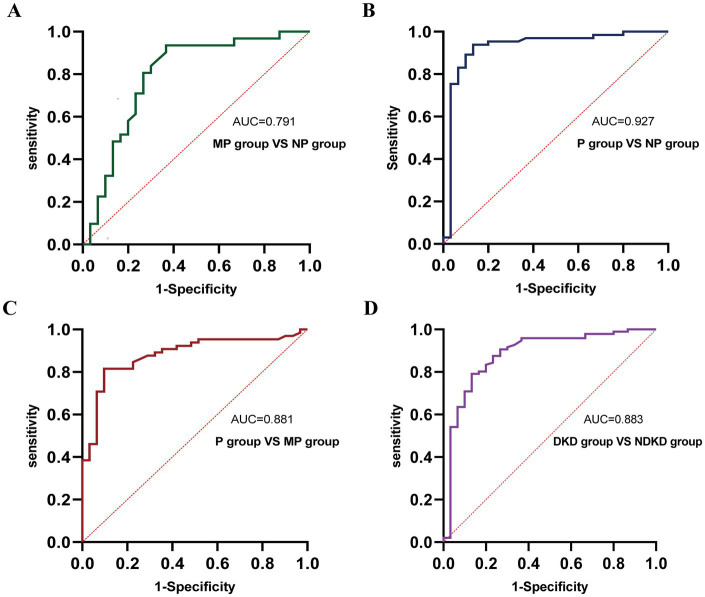
ROC curve analysis of the diagnostic performance of TGF-β1 in different groups. **(A)** MP group vs. NP group. **(B)** P group vs. NP group. **(C)** P group vs. MP group. **(D)** DKD group vs. NDKD group.

**Figure 5:**
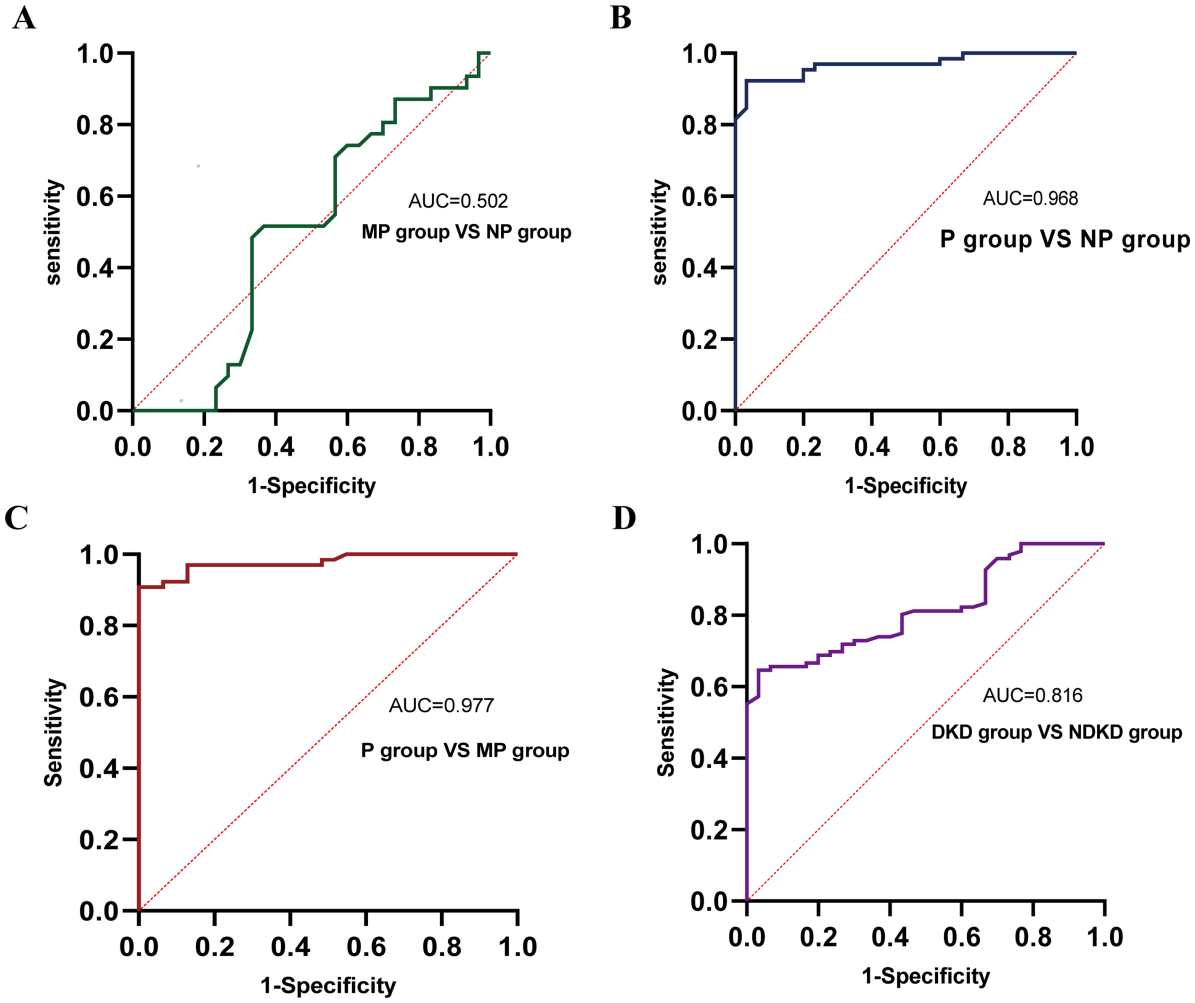
ROC curve analysis of the diagnostic performance of CysC in different groups. **(A)** MP group vs. NP group. **(B)** P group vs. NP group. **(C)** P group vs. MP group. **(D)** DKD group vs. NDKD group.

**Table 2:** Correlation between TGF-β1 and clinical indicators.

Variable	Correlation analysis		Multiple linear regression	
	*r*	*p*-value	*B*	*p*-value
Age (years)	−0.200	0.025	−0.084	0.461
BMI (kg/m^2^)	0.037	0.678	—	—
SBP (mmHg)	0.266	0.003	0.041	0.439
DBP (mmHg)	0.025	0.784	—	—
Hb (g/L)	−0.436	<0.001	0.04	0.517
PLT (10^9^/L)	0.300	0.001	0.051	0.002
FPG (mmol/L)	−0.101	0.259	—	—
UREA (mmol/L)	0.614	<0.001	−0.027	0.934
UA (μmol/L)	0.316	<0.001	−0.002	0.856
TP (g/L)	−0.334	<0.001	0.047	0.750
eGFR (mL/min/1.73m^2^)	−0.611	<0.001	−0.012	0.844
IL6 (pg/mL)	0.226	0.011	0.045	0.708
SII	0.267	0.003	−0.001	0.767
24-UTP (mg)	0.507	<0.001	0.001	0.025
UACR (mg/g)	0.386	<0.001	0.006	0.048

**Table 3:** Correlation between CysC and clinical indicators.

Variable	Correlation analysis		Multiple linear regression	
	*r*	*p*-value	*B*	*p*-value
Age (years)	0.032	0.718	—	—
BMI (kg/m^2^)	0.026	0.770	—	—
SBP (mmHg)	0.253	0.004	0.005	0.821
DBP (mmHg)	−0.164	0.066	—	—
Hb (g/L)	−0.720	<0.001	−0.018	0.448
PLT (10^9^/L)	−0.066	0.465	—	—
FPG (mmol/L)	−0.280	0.002	−0.059	0.416
UREA (mmol/L)	0.864	<0.001	0.261	0.050
UA (μmol/L)	0.487	<0.001	0.007	0.174
TP (g/L)	−0.464	<0.001	0.065	0.278
eGFR (mL/min/1.73m^2^)	−0.888	<0.001	−0.144	<0.001
IL6 (pg/mL)	0.264	0.003	0.009	0.847
SII	0.247	0.005	−5.38 × 10^−6^	0.993
24-UTP (mg)	0.585	<0.001	0.001	0.210
UACR (mg/g)	0.658	<0.001	0.001	0.002

#### 3.1.3 Logistic regression analysis of TGF-β1 and CysC (grouped by proteinuria)

Univariate logistic regression analysis showed that TGF-β1 may be a factor influencing the occurrence of microproteinuria (*p* = 0.003); SBP, Hb, FPG, UREA, UA, TP, Scr, eGFR, IL6, TGF-β1, and CysC may be factors influencing the occurrence of proteinuria (*p* < 0.05) (Supplementary Table S1). After adjusting for factors such as SBP, Hb, FPG, UREA, UA, TP, Scr, eGFR, SII and IL6, it was found that TGF-β1 has been associated with proteinuria levels in T2DM patients. Each unit increase in TGF-β1 was associated with a 1.122-fold and 1.470-fold higher odds of the presence of microalbuminuria and proteinuria, respectively, in the NP group. In contrast, CysC showed no clinical significance after adjustment (see Table 4).

**Table 4:** Multivariate logistic regression analysis of TGF-β1 and CysC.

Group	TGF-β1 (ng/mL)		CysC (10 mg/L)	
	OR (95% CI)	*p*-value	OR (95% CI)	*p*-value
NP group	1		1	
MP group	1.122 (1.028–1.225)	0.010	1.060 (0.769–1.46)	0.721
P group	1.470 (1.049–2.061)	0.025	2.194 (0.765–6.293)	0.144

#### 3.1.4 Logistic regression analysis of serum TGF-β1 and CysC for DKD

The NP group was defined as the NDKD group, while the MP group and P group were defined as the DKD group. Using the presence of DKD as the dependent variable (NDKD = 0, DKD = 1), univariate logistic regression analysis indicated that Hb, UREA, TP, Scr, eGFR, IL-6, TGF-β1, and CysC were potential factors influencing DKD (*p* < 0.05) (Supplementary Table S2). Multivariate logistic regression analysis revealed that, after adjusting for SBP, Hb, FPG, UREA, UA, TP, Scr, eGFR, IL-6, and SII, elevated serum TGF-β1 showed a significant association with DKD. Each unit increase in serum TGF-β1 was associated with a 1.151-fold higher likelihood of DKD occurrence in T2DM patients (Table 5).

**Table 5:** Logistic regression analysis of serum TGF-β1 and CysC for DKD.

Group	TGF-β1 (ng/mL)		CysC (10 mg/L)	
	OR (95% CI)	*p*-value	OR (95% CI)	*p*-value
NDKD group	1		1	1
DKD group	1.151 (1.056–1.254)	0.001	1.052 (0.760–1.458)	0.758

#### 3.1.5 Diagnostic value of serum TGF-β1 and CysC for DKD

**Figure 6:**
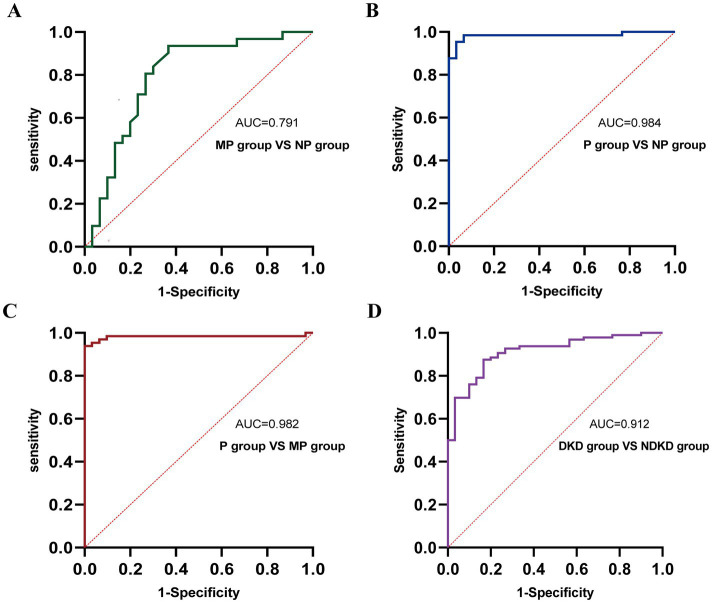
ROC curve analysis of the diagnostic performance of TGF-β1 + CysC in different groups. **(A)** MP group vs. NP group. **(B)** P group vs. NP group. **(C)** P group vs. MP group. **(D)** DKD group vs. NDKD group.

To comprehensively assess the diagnostic potential of TGF-β1 and CysC, we conducted ROC analysis across multiple comparison groups (Figures 4–6 and Table 6). In the MP group versus NP group comparison, TGF-β1 demonstrated an AUC of 0.791, while CysC showed a modest AUC of 0.502, and their combined detection failed to enhance diagnostic accuracy. Notably, in the P group versus NP group, both TGF-β1 (AUC = 0.927) and CysC (AUC = 0.968) exhibited exceptional diagnostic performance, with their combined detection achieving an impressive AUC of 0.984. Similarly, when comparing P group and MP groups, TGF-β1 (AUC = 0.881) and CysC (AUC = 0.977) displayed significant individual diagnostic capabilities, and their combined analysis further elevated the AUC to 0.982. In the DKD versus NDKD group comparison, TGF-β1 yielded an AUC of 0.883, CysC an AUC of 0.816, and their combined analysis resulted in an AUC of 0.912.

**Table 6:** Diagnostic performance of TGF-β1 and CysC across different disease groups.

Group	Index	AUC	*p*-value	95% CI	Optimal cut-off value	Sensitivity	Specificity	Youden index
MP group vs. NP group	TGF-β1	0.791	<0.001	0.671–0.911	21.70 ng/mL	0.936	0.633	0.569
	CysC	0.502	0.977	——	——	——	——	——
	TGF-β1 + CysC	0.791	<0.001	0.671–0.911	——	0.936	0.633	0.569
P group vs. NP group	TGF-β	0.927	<0.001	0.858–0.997	29.10 ng/mL	0.939	0.867	0.805
	CysC	0.968	<0.001	0.936–0.999	14.45 (10 mg/L)	0.923	0.9667	0.890
	TGF-β1 + CysC	0.984	<0.001	0.959–1.000	——	0.954	0.967	0.921
P group vs. MP group	TGF-β1	0.881	<0.001	0.810–0.952	35.43 ng/mL	0.815	0.903	0.719
	CysC	0.977	<0.001	0.953–1.000	14.15 (10 mg/L)	0.923	0.936	0.859
	TGF-β1 + CysC	0.982	<0.001	0.953–1.000	——	0.939	1.000	0.939
DKD vs. NDKD	TGF-β1	0.883	<0.001	0.806–0.960	29.10 ng/mL	0.793	0.867	0.658
	CysC	0.816	<0.001	0.743–0.889	14.45 (10 mg/L)	0.646	0.9667	0.613
	TGF-β1 + CysC	0.912	<0.001	0.858–0.965	——	0.875	0.8333	0.708

NP, normal proteinuria; MP, microalbuminuria; P, proteinuria; DKD, diabetic kidney disease; NDKD, no diabetic kidney disease; AUC, area under the curve; 95% CI, 95% confidence interval.

### 3.2 Analysis based on eGFR groups

#### 3.2.1 Baseline characteristics of patients by eGFR levels

**Figure 7:**
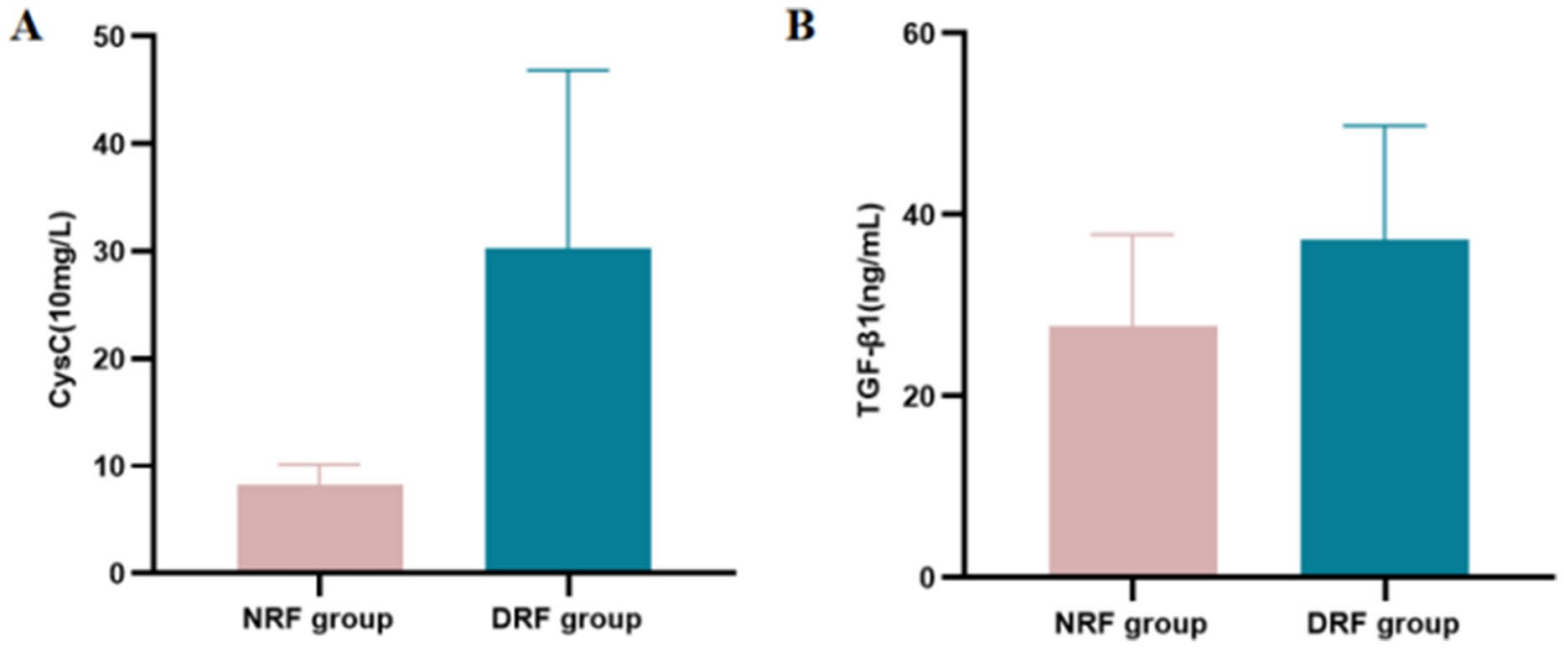
Expression levels of CysC and TGF-β1 in different eGFR groups. **(A)** CysC. **(B)** TGF-β1.

Patients were divided into the NRF group (*n* = 44) and the DRF group (*n* = 82) based on eGFR. No significant differences were observed between the two groups in terms of gender and BMI. The DRF group had significantly higher age and SBP compared to the NRF group, while DBP, Hb, and TP were significantly lower in the DRF group (*p* < 0.05). In terms of metabolic indicators, the DRF group had higher UA levels and lower FPG levels compared to the NRF group (*p* < 0.05). Regarding renal function and proteinuria, the DRF group exhibited significantly higher Scr, UREA, 24-UTP, and UACR (*p* < 0.001). In terms of inflammation markers, IL-6 and SII were significantly elevated in the DRF group (*p* < 0.01) (Table 7). CysC levels were significantly elevated in the DRF group (30.320 ± 16.550 vs. 8.318 ± 1.830 (10 mg/L), *p* < 0.001) (Figure 7A). TGF-β1 was significantly elevated in the DRF group (37.249 ± 12.569 vs. 27.663 ± 10.138 ng/mL, *p* < 0.001) (Figure 7B).

**Table 7:** Comparison of baseline characteristics of patients grouped by eGFR.

Variable	NRF group (*n* = 44)	DRF group (*n* = 82)	*Z*/*χ*^2^/*t*	*p*-value
Age (years)	54.98 ± 10.456	61.93 ± 11.027	3.433	0.001
Gender			1.299	0.254
Male (%)	25 (56.82)	55 (67.07)		
Female (%)	19 (43.18)	27 (32.93)		
BMI (kg/m^2^)	26.157 ± 3.795	25.621 ± 3.627	−0.778	0.438
SBP (mmHg)	135 (115, 144)	143.5 (130.25, 155)	−3.077	0.002
DBP (mmHg)	81.480 ± 13.941	76.700 ± 11.331	−2.081	0.040
Hb (g/L)	143.570 ± 21.118	112.490 ± 23.667	−7.29	<0.001
PLT (10^9^/L)	241 (196, 276)	223 (195, 252.25)	−1.269	0.204
FPG (mmol/L)	10.53 (7.83, 15.82)	9.6 (7.04, 12.445)	−2.835	0.005
UREA (mmol/L)	5.49 (4.8125, 6.4025)	13.305 (7.875, 23.9)	−7.152	<0.001
UA (μmol/L)	318.368 ± 78.615	414.757 ± 89.782	5.992	<0.001
TP (g/L)	70.777 ± 6.743	64.085 ± 8.031	−4.706	<0.001
Scr (μmol/L)	60.7 (51.9, 68.6)	97.95 (79.4, 167.65)	−8.595	<0.001
eGFR (mL/min/1.73m^2^)	103.7611 (96.893, 116.501)	58.878 (33.767, 75.285)	−9.232	<0.001
IL6 (pg/mL)	3.06 (1.5, 6.87)	3.855 (2.545, 6.0725)	−3.085	0.002
SII	491.167 (341.667, 715.826)	548.084 (427.025, 928.193)	−2.83	0.005
CysC (10 mg/L)	8.1 (7.0, 9.7)	17.4 (12.05, 24.45)	−8.751	<0.001
24-UTP (mg)	138 (70, 212)	1,002 (171.25, 3,920)	−7.039	<0.001
UACR (mg/g)	80 (15, 150)	550 (85, 800)	−4.756	<0.001
TGF-β1 (ng/mL)	27.663 ± 10.138	37.249 ± 12.569	4.354	<0.001

BMI, body mass index; SBP, systolic blood pressure; DBP, diastolic blood pressure; WBC, white blood cell count; Hb, hemoglobin; PLT, platelet count; FPG, fasting plasma glucose; UA, uric acid; TP, total protein; Scr, serum creatinine; eGFR, estimated glomerular filtration rate; IL6, interleukin-6; SII, systolic index of inflammation; CysC, cystatin C; 24-UTP, 24-h urinary total protein; UACR, urine albumin creatinine ratio; TGF-β1, transforming growth factor beta 1.

#### 3.2.2 Univariate and multivariate logistic regression analysis of TGF-β1 and CysC

The presence or absence of renal function decline was set as the dependent variable (NRF = 0, DRF = 1), with age, SBP, DBP, Hb, FPG, UREA, UA, TP, IL6, SII, 24-UTP, UACR, CysC, and TGF-β1 as independent variables. Univariate logistic regression analysis revealed that age, SBP, DBP, Hb, FPG, UREA, UA, TP, 24-UTP, UACR, CysC, and TGF-β1 may be factors contributing to renal function decline (*p* < 0.05). In multivariate analysis, after adjustment, only CysC showed a significant independent association (OR = 2.255, 95% CI: 1.240–4.103, *p* = 0.008), indicating that for every 10-unit increase in CysC, the likelihood of renal function decline in NRF patients increases by 2.255 times (see Table 8).

**Table 8:** Univariate and multivariate logistic regression analysis of TGF-β1 and CysC.

Independent variable	Univariate		Multivariate	
	OR (95% CI)	*p*-value	OR (95% CI)	*p*-value
Age (years)	1.059 (1.022–1.098)	0.002	1.253 (0.995–1.579)	0.056
SBP (mmHg)	1.034 (1.012–1.056)	0.002	0.988 (0.916–1.066)	0.759
DBP (mmHg)	0.968 (0.938–0.999)	0.043	1 (0.897–1.115)	1.000
Hb (g/L)	0.943 (0.923–0.964)	<0.001	1.014 (0.923–1.115)	0.769
FPG (mmol/L)	0.933 (0.868–1.002)	0.057	0.964 (0.754–1.232)	0.769
UREA (mmol/L)	1.743 (1.359–2.235)	<0.001	0.837 (0.437–1.602)	0.590
UA (μmol/L)	1.014 (1.008–1.02)	<0.001	1.012 (0.994–1.03)	0.203
TP (g/L)	0.886 (0.836–0.939)	<0.001	1.053 (0.905–1.225)	0.503
IL6 (pg/mL)	1.044 (0.988–1.104)	0.122	0.903 (0.746–1.094)	0.298
SII	1.000 (1.000–1.001)	0.229	1.001 (0.999–1.004)	0.289
24-UTP (mg)	1.002 (1.001–1.002)	<0.001	1 (0.999–1.001)	0.971
UACR (mg/g)	1.005 (1.002–1.007)	<0.001	1.004 (0.999–1.009)	0.098
CysC (10 mg/L)	2.489 (1.626–3.809)	<0.001	2.255 (1.240–4.103)	0.008
TGF-β1 (ng/mL)	1.072 (1.035–1.11)	<0.001	0.934 (0.81–1.078)	0.350

#### 3.2.3 Diagnostic value of serum TGF-β1 and CysC in predicting renal function decline in T2DM patients

**Figure 8:**
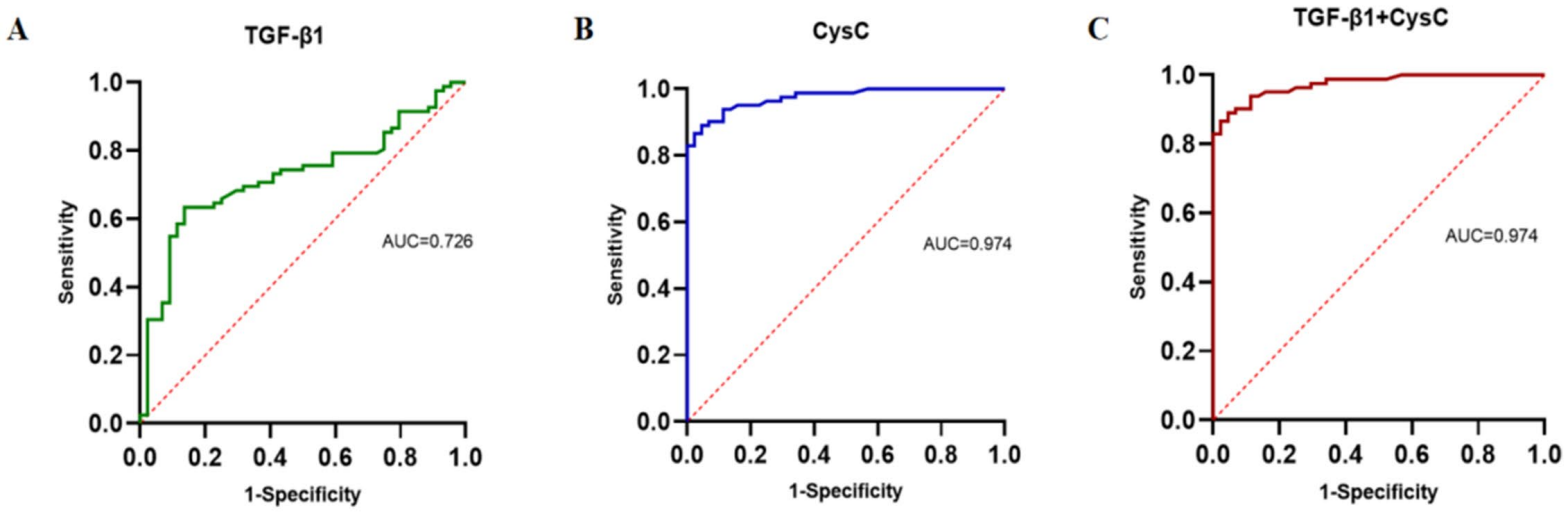
ROC curve for predicting renal function decline. **(A)** TGF-β1. **(B)** CysC. **(C)** TGF-β1 + CysC.

The AUC for using TGF-β1 alone to predict renal function decline was 0.726, suggesting it has some diagnostic value. The AUC for CysC alone was 0.974, demonstrating significantly superior diagnostic performance, with an optimal cutoff value of 11.55 (10 mg/L), corresponding to a sensitivity of 0.89 and specificity of 0.955. The combined detection of TGF-β1 and CysC yielded an AUC similar to that of CysC alone, indicating that combined testing did not further enhance diagnostic performance. In summary, CysC alone demonstrated significantly superior diagnostic efficacy in predicting renal function decline compared to TGF-β1, and combined testing had a similar effect to CysC alone. This suggests that CysC can serve as an important marker for predicting renal function decline (see Figure 8 and Table 9).

**Table 9:** Diagnostic performance of TGF-β1 and CysC in predicting renal function decline.

Index	AUC	*p*-value	95% CI	Optimal cut-off value	Sensitivity	Specificity	Youden index
TGF-β1	0.726	<0.001	0.637–0.815	35.43 ng/mL	0.634	0.864	0.498
CysC	0.974	<0.001	0.952–0.996	11.55 (10 mg/L)	0.890	0.955	0.845
TGF-β1 + CysC	0.974	<0.001	0.952–0.996	—	0.890	0.955	0.845

AUC, area under the curve; 95% CI, 95% confidence interval.

## 4 Discussion

**Figure 9:**
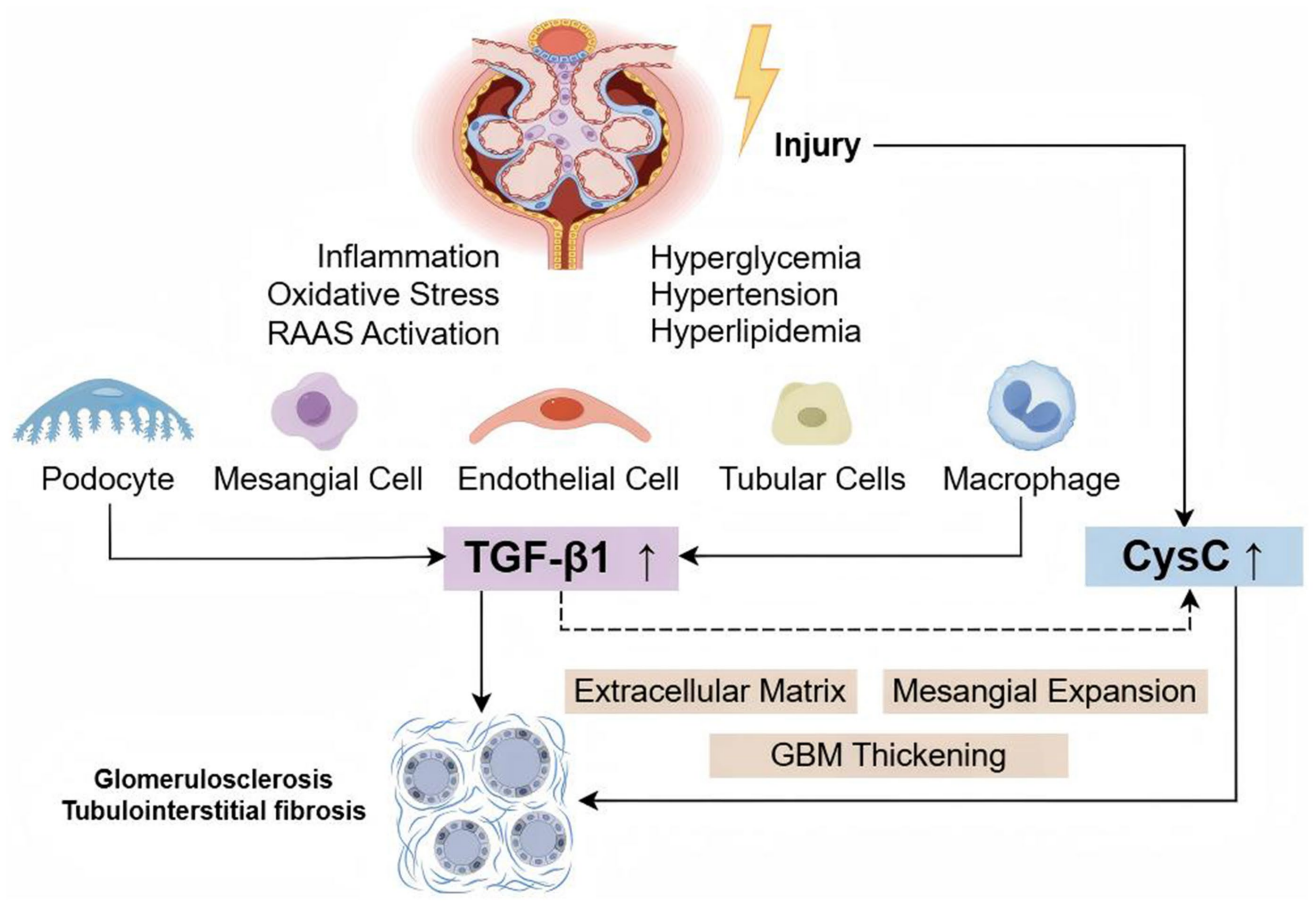
TGF-β1 and CysC in the pathogenesis of DKD. Hyperglycemia, hypertension, hyperlipidemia, inflammation, oxidative stress, and other factors can damage renal cells (podocytes, mesangial cells, endothelial cells, tubular cells, and macrophages), upregulating the expression of TGF-β1 and CysC. TGF-β1 promotes CysC production, and together, they contribute to extracellular matrix accumulation, mesangial expansion, and thickening of the glomerular basement membrane, ultimately leading to glomerulosclerosis and tubulointerstitial fibrosis.

DKD is characterized by thickening of the glomerular basement membrane, mesangial matrix proliferation, interstitial fibrosis, and chronic inflammatory responses, leading to gradual renal dysfunction. TGF-β1 plays a key regulatory role in these processes (13, 25–27). CysC is a cysteine protease inhibitor expressed and secreted by all nucleated cells, which is ultimately cleared by the kidneys. Serum CysC is a sensitive marker of both acute and chronic renal function changes (28, 29). Cysteine protease is a key substance involved in the recycling and remodeling of basement membrane and extracellular matrix components (30). Studies have shown that dysregulation of CysC and cysteine proteases plays an important pathological role in fibrosis, and CysC may serve as an effective biomarker for organ fibrosis (18). TGF-β1 promotes the production and secretion of CysC (31, 32) (Figure 9). Therefore, this study explored the diagnostic value of TGF-β1 and CysC in patients with DKD and their correlation with clinical indicators. The research findings indicate that these two biomarkers have significant value in the early diagnosis and disease assessment of DKD.

### 4.1 Diagnostic value of TGF-β1

Serum TGF-β1 has been associated with proteinuria levels in T2DM patients. TGF-β1 levels show a significant positive correlation with proteinuria severity, consistent with previous studies (33, 34). Multivariate logistic regression analysis reveals that each unit increase in TGF-β1 was associated with a 1.122-fold and 1.470-fold higher odds of the presence of microalbuminuria and proteinuria, respectively, in the NP group. TGF-β1 demonstrates good diagnostic performance for DKD and different degrees of proteinuria. Impaired resolution of inflammation is a major driver of DKD progression (35). We found that TGF-β1 is significantly positively correlated with inflammatory markers of DKD, such as IL-6 and SII (36). In studies using the LLC-PK1 cell model of DKD, the protective effect of the antidiabetic drug liraglutide may be associated with the inhibition of TGF-β1, and further *in vitro* and *in vivo* studies are needed to clarify its specific mechanism of action (37). Collectively, these findings suggest that TGF-β1 may serve as a biomarker for DKD, providing predictive insights into the progression of kidney disease. Similarly, previous studies have confirmed that serum TGF-β1 levels are also correlated with renal function in other types of CKD (such as IgA nephropathy, hypertensive nephropathy) (38–41). Its diagnostic efficacy in different kidney diseases warrants further investigation.

### 4.2 Diagnostic value of CysC

This study demonstrated a significant association between serum CysC levels and renal function decline in T2DM patients. Each 10-unit elevation in CysC concentration was associated with a 2.255-fold increased likelihood of renal deterioration. ROC curve analysis demonstrated an AUC of 0.974 for CysC in diagnosing renal function decline in T2DM patients, with a sensitivity of 91.5%, consistent with findings from Ren et al. (17). Additionally, CysC showed a significant positive correlation with proteinuria. However, ROC curve analysis revealed an AUC of only 0.502 for CysC in distinguishing MP from NP groups, indicating limited efficacy in diagnosing microalbuminuria. This is consistent with the study by Visinescu et al. (42): there was no significant difference in serum CysC levels between the normal albuminuria group and the microalbuminuria group. This phenomenon may be attributed to the relatively short and similar duration of diabetes (10–12 years) in the two groups of subjects in this study. It is known that a longer duration of diabetes is a factor contributing to increased CysC levels, which may lead to kidney damage. Moreover, a review of previous literature suggests that fluctuations in CysC levels may not solely reflect renal function (43). And in clinical practice, it is often used in conjunction with serum creatinine to assess GFR (44). However, its limitations should be carefully considered: corticosteroid treatment and abnormal thyroid hormone levels (particularly subclinical thyroid dysfunction) can cause significant fluctuations in CysC concentrations (28, 45). It is noteworthy that there is a significant comorbid relationship and bidirectional pathophysiological association between thyroid dysfunction and chronic kidney disease (46). In terms of detection technology, there is currently no standardized reference system for CysC testing internationally, and the bias in testing systems poses challenges to the clinical interpretation of single CysC measurements. Continuous dynamic monitoring is necessary to improve the reliability of results (44, 47–50). In contrast, CysC demonstrated good diagnostic performance for identifying proteinuria. This finding aligns with the results of Li et al. (51), suggesting that CysC may not be an effective biomarker for early DKD detection. However, numerous studies have shown that serum CysC is more sensitive than albuminuria in early DKD, and equations using CysC for eGFR calculation outperform those using creatinine (42, 52, 53). For diagnosing NDKD and DKD, CysC yielded an AUC of 0.816. While lower than that of TGF-β1, it still exhibited good diagnostic efficacy. This suggests that combined diagnostic approaches may enhance the accuracy of disease evaluation.

### 4.3 Clinical application of TGF-β1 and CysC

In the clinical management of DKD, the establishment of early evaluation is of significant importance for delaying disease progression. In the assessment of DKD, both CysC and TGF-β1 have their respective advantages in application. TGF-β1 is associated with microalbuminuria and inflammatory markers, showing promise in the early detection of DKD. CysC is more significantly elevated when renal function declines, further reinforcing the current KDIGO guidelines recommending the use of CysC for eGFR in diabetes (54). The diagnostic efficacy of combined assessment of CysC and TGF-β1 is superior to or at least equal to that of individual assessments, with the combined testing strategy demonstrating outstanding ability in distinguishing patients with severe proteinuria. Future efforts should focus on further optimizing the combined diagnostic model, incorporating dynamic monitoring and stratified management, to enhance its clinical feasibility and accuracy. Moreover, its utility in longitudinal studies should be evaluated, providing a more comprehensive diagnosis and treatment plan for DKD patients.

## 5 Limitations

This study has several limitations: (1) The sample size is relatively limited, and a more detailed stratified analysis could not be performed. (2) The study did not include non-diabetic CKD patients as a control group, which somewhat limits the applicability of the findings. (3) The cross-sectional design limits the ability to infer causality and may introduce confounding factors that could affect the results. (4) Although key clinical variables were adjusted, the potential impact of other factors, such as the treatment with renin-angiotensin-aldosterone system (RAAS) inhibitors and sodium-glucose cotransporter 2 (SGLT2) inhibitors, on biomarker levels could not be fully excluded. Future studies should expand the sample size, conduct multicenter research, and design studies with multiple control groups (including healthy control group, non-diabetic CKD group, and diabetic nephropathy group) to validate the disease specificity of the biomarkers and enhance the generalizability of the findings. Longitudinal study designs should be adopted, with multiple follow-ups and data collection at different time points, enabling the analysis of the trend of variable changes over time.

## 6 Conclusion

This study confirms the diagnostic value of CysC and TGF-β1 in DKD. The combined detection of both biomarkers provides new methods for early diagnosis, disease monitoring, and prognosis evaluation of DKD. However, these findings still require further research to validate and refine.

## Data Availability

The raw data supporting the conclusions of this article will be made available by the authors, without undue reservation.
