# Supplementary material for: Diagnostic value of serum TGF-β1 and CysC in type 2 diabetic kidney disease: a cross-sectional study
Source: Front Med (Lausanne). 2025 Apr 11;12:1529648. doi: 10.3389/fmed.2025.1529648 (PMC12021808; doi:10.3389/fmed.2025.1529648)
Supplement: Supplementary file 1 [file Table_1.DOCX]

**Supplementary material**

**1 TGF-β1 measurement**

The kit utilizes a double antibody sandwich ELISA method. In this assay, the anti-TGF-β1 antibody is immobilized on a microplate, and during the experiment, TGF-β1 in the sample (or standard) binds to the immobilized antibody. Subsequently, biotinylated anti-TGF-β1 antibody and horseradish peroxidase-conjugated streptavidin are added. The anti-TGF-β1 antibody binds to the TGF-β1 immobilized on the coated antibody, and biotin binds specifically with streptavidin to form an immune complex. Any unbound components are washed away. Afterward, a colorimetric substrate (TMB) is added, which turns blue under the catalysis of horseradish peroxidase and changes to yellow upon the addition of the stop solution. The OD value at 450 nm is measured using a microplate reader, and the TGF-β1 concentration is directly proportional to the OD450 value. The concentration of TGF-β1 in the sample is determined by plotting a standard curve.

**2 Logistic regression analysis of TGF-β1 and CysC (grouped by proteinuria)**

**Table S1 Univariate logistic regression (grouped by proteinuria).**

| Independent variable | MP group | | P group | |
| --- | --- | --- | --- | --- |
|  | OR(95%CI) | *P* value | OR(95%CI) | *P* value |
| SBP(mmHg) | 0.993(0.967-1.019) | 0.584 | 1.027(1.003-1.052) | 0.028 |
| Hb(g/L) | 0.986(0.96-1.014) | 0.322 | 0.909(0.879-0.94) | <0.001 |
| FPG(mmol/L) | 0.969(0.89-1.054) | 0.460 | 0.916(0.842-0.997) | 0.043 |
| UREA(mmol/L) | 0.975(0.741-1.282) | 0.855 | 1.909(1.426-2.554) | <0.001 |
| UA(μmol/L) | 0.997(0.991-1.003) | 0.270 | 1.008(1.003-1.013) | 0.003 |
| TP(g/L) | 0.992(0.918-1.072) | 0.836 | 0.836(0.774-0.903) | <0.001 |
| Scr(μmol/L) | 0.996(0.966-1.026) | 0.780 | 1.078(1.038-1.121) | <0.001 |
| eGFR(ml/min/1.73m^2^) | 1.018(0.99-1.046) | 0.203 | 0.908(0.873-0.945) | 0.001 |
| IL6(pg/mL) | 1.077(0.958-1.21) | 0.213 | 1.129(1.012-1.259) | 0.029 |
| SII | 0.999(0.998-1.001) | 0.266 | 1.000(1.000-1.001) | 0.282 |
| CysC(10mg/L) | 0.979(0.819-1.169) | 0.811 | 1.617(1.293-2.024) | <0.001 |
| TGF-β1(ng/mL) | 1.108(1.035-1.186) | 0.003 | 1.292(1.187-1.407) | <0.001 |

**3 Logistic regression analysis of serum TGF-β1 and CysC for DKD**

**Table S2. Logistic regression analysis of serum TGF-β1 and CysC for DKD**

| Independent variable | Univariate | | Multivariate | |
| --- | --- | --- | --- | --- |
|  | OR(95%CI) | *P* value | OR(95%CI) | *P* value |
| SBP(mmHg) | 1.014(0.993-1.036) | 0.186 | 0.999(0.966-1.034) | 0.967 |
| Hb(g/L) | 0.951(0.929-0.972) | <0.001 | 0.962(0.916-1.01) | 0.102 |
| FPG(mmol/L) | 0.937(0.871-1.008) | 0.082 | 0.978(0.863-1.108) | 0.727 |
| UREA(mmol/L) | 1.364(1.141-1.631) | 0.001 | 1.101(0.769-1.576) | 0.600 |
| UA(μmol/L) | 1.004(1.000-1.009) | 0.079 | 0.999(0.991-1.007) | 0.793 |
| TP(g/L) | 0.898(0.844-0.956) | 0.001 | 0.96(0.875-1.054) | 0.396 |
| Scr(μmol/L) | 1.028(1.008-1.048) | 0.006 | 1.027(0.962-1.096) | 0.423 |
| eGFR(ml/min/1.73m^2^) | 0.968(0.953-0.982) | <0.001 | 1.03(0.976-1.087) | 0.282 |
| IL6(pg/mL) | 1.113(1.001-1.237) | 0.048 | 1.046(0.944-1.159) | 0.395 |
| SII | 1.000(1.000-1.001) | 0.475 | 1.000(0.999-1.001) | 0.364 |
| CysC(10mg/L) | 1.207(1.083-1.345) | 0.001 | 1.052(0.760-1.458) | 0.758 |
| TGF-β1(ng/mL) | 1.175(1.104-1.251) | <0.001 | 1.151(1.056-1.254) | 0.001 |
